# Anti-Black racism in Canadian health care: a qualitative study of diverse perceptions of racism and racial discrimination among Black adults in Montreal, Quebec

**DOI:** 10.1186/s12889-024-20636-0

**Published:** 2024-11-13

**Authors:** Khandideh K. A. Williams, Shamara Baidoobonso, Aisha Lofters, Jeannie Haggerty, Isabelle Leblanc, Alayne M. Adams

**Affiliations:** 1 Department of Family Medicine, McGill University, Montreal, Canada; 2 St. Mary’s Research Center, St. Mary’s Hospital Center, Montreal, Canada; 3 Department of Community Health & Epidemiology, Dalhousie University, Halifax, Canada; 4 Department of Family & Community Medicine, University of Toronto, Toronto, Canada; 5 Peter Gilgan Centre for Women’s Cancers, Women’s College Hospital, Toronto, Canada; 6 University Family Medicine Group St. Mary, St. Mary’s Hospital Center, Montreal, Canada

**Keywords:** Racism, Racial discrimination, Health care, Health care equity, Anti-black racism, Black health, Intersectionality, Montreal, Quebec, Canada

## Abstract

**Background:**

Racism has been shown to impact the health of Black persons through its influence on health care, including its expression through implicit biases in provider training, attitudes, and behaviours. Less is known about the experiences of racism in contexts outside of the USA, and how race and racism interact with other social locations and systems of discrimination to shape Black patients’ experiences of racism in health care encounters. To help address this gap, this study examined diverse Black individuals’ perceived experiences of, and attitudes towards, anti-Black racism and racial discrimination in Canadian health care, specifically in Montreal, Quebec.

**Methods:**

This descriptive qualitative study adopted a social constructionist approach. Employing purposive maximal variation and snowball sampling strategies, eligible study participants were: self-identified Black persons aged 18 years and older who lived in Montreal during the COVID-19 pandemic, who could speak English or French, and who were registered with the Quebec medical insurance program. In-depth interviews were conducted, and a Framework Analysis approach guided the systematic exploration and interpretation of data using an intersectionality lens.

**Results:**

We interviewed 32 participants, the majority of whom were women (59%), university educated (69%), and modestly comfortable financially (41%), but diverse in terms of age (22 to 79 years), country of origin, and self-defined ethnicity. We identified five major themes demonstrating substantial variations in perceived racism in health care that are influenced by unique social locations such as gender identity, age, and immigration history: (1) no perceptions of racism in health care, (2) ambiguous perceptions of racism in health care, (3) perceptions of overt interpersonal racism in health care, (4) perceptions of covert interpersonal racism in health care (including the downplaying of health concerns, stereotyping, and racial microaggressions), and (5) perceptions of systemic racism in health care.

**Conclusions:**

Perceptions of anti-Black racism and racial discrimination in Canadian health care are complex and may include intra-racial group differences. This study begins to address the dearth of empirical research documenting experiences of anti-Black racism in health care in Quebec, highlighting a continued need for serious consideration of the ways in which racism may manifest in the province, as well as a need for anti-racist advocacy. Advancing racial health equity requires greater sensitivity from providers and decision makers to variations in Black patients’ health care experiences, towards ensuring that they have access to high quality and equitable health care services.

**Supplementary Information:**

The online version contains supplementary material available at 10.1186/s12889-024-20636-0.

## Background

Disproportionate disease burdens experienced by Black populations in the United States have been documented in relation to conditions such as asthma, diabetes, hypertension, and various types of cancers [1–6]. For example, although breast cancer incidence rates are highest among White women, Black women are among those with the highest breast cancer-specific mortality rates [1]. In 2018, African Americans were 50% more likely to have a stroke and African American men, specifically, were 70% more likely to die from a stroke, in comparison to their non-Hispanic White counterparts [7]. Overall, Black populations in the USA experience lower life expectancies, with the 2020 national US data indicating a 5.8-year differential comparing Black and non-Hispanic White groups (71.8 years vs. 77.6 years) [8]. While the vast majority of this work has been conducted in the USA, similar health disparities have been reported in other regional contexts from the UK to the Latin Americas [9–11]. Similar trends have also been observed in Canada [12–15]. For example, one study reported that Black Canadians were significantly more likely to report diabetes and hypertension [16]. Another recent study reported increased cause-specific mortality rates among Black individuals in Canada for conditions such as HIV/AIDS, prostate cancer, diabetes, and hypertension, when compared to their White counterparts [17].

The factors that produce and sustain such racial health disparities are complex and multi-dimensional in nature and are shaped by inequitable social processes such as racism and racial discrimination. A large body of research has documented how racism and racial discrimination impact the physical and mental health of Black groups due to, for example, negative psychological and physiological stress responses, engagement in risky health behaviours, and inequitable access to socioeconomic resources and opportunities [18–21]. Investigations have also shown that various, interrelated forms or levels of racism may drive racial health disparities through their impact on health care. At the micro or interpersonal level, racism can be manifested in biased attitudes and behaviours among health care providers [18, 22–24]. In these contexts, patient interactions with health care professionals may be characterized by microaggressions, prejudice, and racial discrimination, leading to disparate care. Structural (or systemic) racism operates at the macro level and is characterized by the abstract foundational workings of society that produce inequitable access to and advantage from society’s resources, whether financial, material, political or social [25, 26]. It carries the legacies of colonialism and race-based slavery and refers to the patterned ways in which ideas and images are propagated across social contexts (including health care) to produce racialized hierarchies [25, 26]. Examples of the ways in which structural racism may impact health care include inequities in health insurance coverage [27], through the patterned neglect of communities in health care [28, 29], and through racial framing that reifies racial stereotypes, racist ideologies, narratives and representations, and racialized hierarchies [30]. For example, Hoffman and colleagues found that the endorsement of medical trainees’ false beliefs of biological differences between Black and White populations are associated with racial biases in pain assessments, with lower pain scores and reduced accuracy of treatment recommendations being reported at higher frequencies in Black versus White populations [31]. Here, medical trainees internalized master narratives concerning racialized pain tolerance, hindering the care that they provided to Black patients. Institutional racism operates at the meso level, between the structural (macro level) and interpersonal (micro level) racisms and refers to the routine manifestation of racism through practices, policies and procedures across an organization/institution [25]. These dimensions of racism interact with one another in complex ways through individual interactions, institutional practices and procedures, and societal norms and imageries that reify racialized experiences in diverse social arenas, including health care.

Perceived racial discrimination in health care has been associated with poorer self-reported health, lower perceived quality of care, unmet needs for care, and delays in seeking care [24, 32–34]. The literature has also identified different ways in which racism is perceived and experienced among Black groups in health care, such as through provider insults, dismissal, or scolding [35, 36]. Still, important research gaps remain surrounding the investigation of perceptions and experiences of racism in health care contexts outside of the USA. Similar to the dearth of Canadian racial health disparities literature, the evidence base regarding racial disparities in health care in Canada remains nascent despite increasing concerns about racial health disparities [37–40]. These concerns were heightened during the COVID-19 pandemic, with indications that Black populations were overrepresented in COVID-19 infection and mortality rates across the country [41, 42]. In the post-pandemic period, an emerging body of work is interrogating the role of racism in producing racial health disparities among Black populations in Canada [43, 44]. However, the manifestation and influence of anti-Black racism within the context of health care encounters is relatively under-investigated. The need for research that explicates how perceptions of racism and racial discrimination impact health care attitudes and experiences among Canada’s Black population is clear and increasingly recognized [45, 46].

This study aims to examine diverse Black individuals’ perceived experiences of, and attitudes towards, anti-Black racism and racial discrimination in Canadian health care, particularly in Montreal, Quebec. In this paper, “racism” is defined as “a societal system built by a dominant racial group based on a belief of their intrinsic superiority, which categorizes individuals into “races” and uses collective power to structure society in a way that provides unearned advantages to the dominant race whilst creating structural disadvantages for non-dominant races deemed inferior” [47]. We acknowledge the historical, political, and structural dimensions of racism in Canada, recognizing its roots stemming from the institution of slavery that produced a racial logic underpinned by ideologies of White superiority that classifies racialized minorities as inherently inferior [48, 49]. We also acknowledge the context-specific dimension of racism, recognizing that its expression in the province of Quebec has adapted with time and is unique to the rest of Canada, largely owing to its position as a national minority vis a vis its dominant French-Canadian population and ensuing Quebec nationalism [48]. “Race” is understood as a social construct embedded in historical, political, and economic realities which assigns social identities and stereotypical attributes to human groups, leading to real-world consequences along social, material, and political dimensions. Similarly to our conceptualization of “racism”, we also acknowledge the dynamic features of “race”, recognizing that the ways in which individuals have been racialized in Canada has been remapped in relation to historical and political contexts [50]. “Anti-Black racism” refers to the unique manifestation of racism directed towards Black individuals, and “perceived racial discrimination” is used to describe perceptions of differential treatments of groups designated as Black that may be a consequence of racial prejudice (negative attitudes and beliefs) produced by anti-Black racism.

## Methods

### Study design and setting

This paper is nested in a larger qualitative, phenomenology [51, 52] study that examines perceptions of racial discrimination in health care and its impacts on accessibility and quality of care among Black communities before and during the COVID-19 pandemic. It draws on data generated via a qualitative descriptive methodology [53, 54] that elicits rich and detailed insights about participants’ experiences with health care services. We adopted a social constructionist perspective which contends that knowledge and truth are socially constructed and understood through individuals’ interactions and experiences within society, including through institutional images and practices [55].

We conducted this study in Montreal, Quebec, one of Canada’s most ethno-culturally diverse cities. In 2021, around 27% of Montreal residents self-identified as a visible minority, with the city’s Black population accounting for the largest visible minority group [56, 57]. Projections indicate that Montreal’s Black population will continue to grow in the coming years and, by 2041, will remain the city’s largest visible minority group comprising over 30% of the total visible minority population [58].

This study must further be contextualized within the context of a Canadian healthcare system and within the unique racial fabric of Quebec. First, Canada’s publicly funded healthcare system (Medicare) is primarily managed, organized, and delivered by the country’s ten provinces and three territories under individual insurance plans [59]. The federal government is in turn responsible for setting and administering national standards via the Canada Health Act, and for providing support with funding and the delivery of health care services to particular population groups (e.g., First Nation communities living on reserves, Inuit, and members serving in the Canadian Forces) [59]. This system aims to provide reasonable access to *necessary* medical care to all Canadian residents, without out-of-pocket fees. Quebec’s health and social services system, specifically, is designed to provide access to various integrated and coordinated health and social services which are divided into service programs aimed at providing care to the general population (e.g., public health, frontline clinical services and social assistance programs for temporary concerns) and support programs aimed at responding to specific needs (e.g., support for independent seniors, physical disabilities and impairments, mental health, and physical health including emergency and specialized services) [60].

It is also worth noting that this study is embedded within Quebec’s distinct racial discourses and racial relations produced by the province’s unique sociopolitical history. The development of the province’s “nation within a nation” identity following British conquest and colonization in 1760 led to two centuries of marginalization, oppression and economic exploitation of French Canadians’ language, culture, and identity by the dominant British minority [48, 61]. By the 1960s, a period of political and social disruption referred to as the Quiet Revolution, these deprived conditions led many French-Canadians to compare their social conditions to the plight of African Americans, appropriating the Black civil rights struggle and invoking notions of French Canadians as “nègres” (meaning both “niggers” and “negroes”) [48, 62]. The result was an adapted French-Canadian master narrative that shaped understandings of the province’s colonial history by focusing on the English and the French as the founding “races”, while disavowing French colonization of Indigenous peoples and slavery in New France [63]. Indeed, the enslavement of peoples of African ancestry was institutionalized in Canada during both the French and British colonial regimes, with the first record of an enslaved Black person dating back to 1628 [49]. This master narrative presented the lived experiences of French-Canadians as the “Nègres blancs d’Amérique” (popularized through Pierre Vallières best-selling book, later translated to “White Niggers of America”), while denying the presence of racism faced by Quebec’s (and specifically, Montreal’s) small but growing Black population [48, 62]. This narrative was further engendered by a strong sense of Quebec nationalism that amplified during the 1960s and which may be characterized, among other things, by a sense of ethnic nationalism (e.g., as seen through the adoption of notions suggesting the racial purity of French-Canadians, such as “pure laine” – literally meaning pure wool), and linguistic nationalism (which privileged the French language in public spaces such as schools and workplaces and, more recently, health care) [61, 64]. Racial identity and racism in Quebec are thus complicated by a colonial history that made French Quebecers a cultural and linguistic minority in North America. This unique position, as well as national narratives that deny Canada’s history of colonial violence and promote ideas of the country’s purported racial neutrality, perpetuate the marginalization and discrimination of persons who do not fit this dominate narrative, including Black persons [48, 62]. Together, this contributes to a lack of attention paid to anti-Black racism in Quebec, particularly in health care despite several reports indicating why consideration and related action are important [65–67].

### Study participants and recruitment

We purposively selected participants through maximal variation and snowball sampling strategies. Initial recruitment efforts included the dissemination of recruitment posters on the social media pages, websites, and email newsletters of partnering community organizations and academic student associations serving Black, Caribbean and/or African individuals in Montreal. The services provided by these organizations vary and include, for example, activities and programs aimed at improving the quality of life of Black, Caribbean, and/or African people in Montreal. Initial efforts also included the dissemination of recruitment posters in Facebook groups aimed at connecting and empowering Montreal’s Black communities. Secondary recruitment efforts were further facilitated by leaders of partnering community organizations and entailed presenting the call for participants at community events and/or directly approaching members of their organizations by email or telephone. Recruitment posters were later shared on LinkedIn, and participants were also invited to share the recruitment call within their own networks following their participation in the study.

Individuals interested in participating contacted the first author (KKAW) via telephone, email, or social media messaging applications. The first author screened the eligibility of each interested participant via a questionnaire that included questions about their racial identity, self-defined ethnicity, age, sex assigned at birth, gender identity, immigration history, educational attainment, and financial situation. Racial identity here refers to a person’s sense of belonging to a particular racialized group, while self-defined ethnicity refers to a person’s sense of belonging to a particular ethnic group having shared heritage, culture, and language, among other factors. Such characteristics were assessed to facilitate the recruitment of a diverse sample given the heterogeneity of Montreal’s Black population which may impact experiences and perceptions of racism [68]. Eligible participants were those aged 18 years and older who self-identified as Black, lived in Montreal during the COVID-19 pandemic, were able to communicate in French or English, and were registered with the Quebec medical insurance program. Individuals not registered with the provincial medical insurance program were excluded from the study as the absence of health insurance coverage has been documented to create additional barriers to accessing health care that were not being explored in this study.

### Data collection

Data were collected via semi-structured in-depth telephone interviews, allowing for participants to recount their experiences and how they interpreted them in their own words while also abiding by Montreal’s social distancing policies implemented during the peak of the COVID-19 pandemic [69]. In-person interviews were also offered in case participants did not own a telephone. However, all participants indicated a preference for a telephone interview.

The interview guide (see Additional File 1) used to inform the interviews’ themes was developed collaboratively by KKAW, AMA, and JH, with feedback from SB, AL, and IL, and was informed by intersectionality theory. Intersectionality is a theoretical perspective that examines how multiple systems of power interrelate at multiple levels of society to drive advantage, disadvantage, and oppression [70, 71]. More specifically, it elucidates how multiple social locations (such as race, gender identity, income and educational attainment) interact and affect one another at the individual level through interlocking systems of discrimination at the social-structural level (such as racism, sexism, and classism) to shape experiences of inequality [72, 73]. The first part of the interviews asked participants to describe their general experiences with health care in Montreal. The second part focused on whether participants had ever perceived race (i.e., racial dynamics) and/or racism to positively or negatively impact their health care experiences. The final part of the interview asked participants to reflect on possible strategies to redress manifestations of anti-Black racism and racial discrimination in health care and promote health care quality and accessibility for Black communities.

Probing questions were used throughout the interviews to invite participants to reflect on specific moments and actions of varying health care personnel in which racial dynamics and/or racism was an issue, and their reactions and feelings in these situations. During each part of the interview, participants were also prompted to reflect on how their varying social locations might have shaped their experience, and to compare their experiences during the COVID-19 pandemic to their experiences prior to the pandemic. In this paper, we report on our findings related to perceptions of racism and racial discrimination in health care.

Data collection spanned from October 2021 to July 2022. All interviews were conducted by KKAW, a qualitatively trained, Canadian-born, Black-identifying, bilingual, female doctoral student. Interviews were conducted in English, French, or both languages, in accordance with the participants’ language preference. KKAW obtained the participants’ consent verbally prior to commencing the interviews and offered to email a copy of the detailed consent form to each participant. Interviews were audio-recorded, and notes were hand-written by KKAW with the participants’ consent. Following each interview, KKAW transcribed and anonymized each interview recording. The names of participants mentioned in this article are pseudonyms.

### Data analysis

Data were analyzed by KKAW, using Framework Analysis [74, 75], a structured approach to thematic analysis. Reflexive debriefing with AMA occurred at every stage of the analysis process as a means of data verification. Using an intersectionality lens and guided by Jones’ “Gardener’s Tale” theoretical framework of the levels of racism [76], KKAW began by reading and rereading the interviews’ transcripts and notes to gain familiarity. Based on the interview guide, her readings, and her detailed notes, KKAW generated a code book in which she listed and defined a priori and inductive codes. KKAW adapted the codes to reduce redundancies and to improve the clarity of their definitions with AMA’s guidance and feedback. This code book ensured KKAW’s consistent application of the codes across interview transcripts.

Using the data management software Dedoose, each interview transcript was systematically coded. A code report was produced that included all excerpts related to each sub-code within the larger “perceived racism” parent code. KKAW read and reread all excerpts associated to the same group of codes and met with AMA and JH to reflect on and refine emerging themes. To further ascertain their credibility across the sample of participants, KKAW created a data matrix summarizing the narratives of each participant against the broader themes identified (see Additional File 2). She also presented her preliminary findings to members of a partnering community organization, receiving further feedback on their credibility. The data matrix enabled the systematic identification of patterns, adding rigour to analysis, and reifying the credibility of findings and their interpretation.

### Ethics approval

Ethics approval was obtained from the Research Ethics Board of the CIUSSS de l’Ouest-de-l’Île-de-Montréal – biomedical subcommittee at the St. Mary’s Research Centre.

## Results

Thirty-two individuals participated in this study. Eight additional persons expressed interest in participating but were deemed ineligible due to either not being registered with the Québec medical insurance program or not having lived in the city of Montreal during COVID-19. Table 1 summarizes the study participants’ sociodemographic information. The participants were predominantly women (59%), university educated (69%), modestly comfortable financially (41%), and most (63%) preferred to communicate in English rather than French. There was a wide range in age (22 to 79 years) and country of origin. The self-defined ethnicity of each participant represented 20 regions, examples including: Haiti, Jamaica, Trinidad, Nigeria, Mali, Congo and Canada.

**Table 1:** Demographic characteristics of participants ǂ

Characteristic		No. of participants
Age	Range, yr	22–79
	Mean, yr	42
Sex assigned at birth	Female	20 (63%)
	Male	12 (38%)
Gender identity	Woman	19 (59%)
	Man	12 (38%)
	Non-binary	1 (3%)
Immigration history	Canadian-born	16 (50%)
	Immigrant 10 years or less*	6 (19%)
	Immigrant over 10 years◊	10 (31%)
Preferred language of communication	French	9 (28%)
	English	20 (63%)
	Both	3 (9%)
Financial situation	Poor	1 (3%)
	Tight	5 (16%)
	Modestly comfortable	13 (41%)
	Comfortable	9 (28%)
	Prefer not to answer	4 (13%)
Educational Attainment	High school or less	4 (13%)
	College or trade school	6 (19%)
	University Degree	22 (69%)

ǂ All information was self-reported by the study’s participants during eligibility screening

*Immigrated to Canada within the last 10 years

◊Immigrated to Canada over 10 years ago

Each interview lasted between 35 min and 2 h. Participants reported varying degrees of perceived racism in health care, primarily within the context of primary care (e.g., family medicine clinics and community health centers) and emergency departments. Fewer accounts referred to secondary care and even fewer to tertiary care. We generated five major themes relating to perceived experiences of racism in health care, and the ways in which social locations such as age, gender identity, and immigration history intersect to influence said experiences:

### Theme 1. No perceptions of racism in health care

Participants’ personal experiences with race relations, including positive or negative interracial encounters and time spent in race-conscious societies, influenced their perceptions about racism in health care. After reflecting on inter- and intra-racial experiences in their everyday lives, some participants recounted having never perceived racism during their health care encounters compared to other contexts, such as in their places of employment or in health care systems outside of Canada. This occurrence was particularly reflected in the narratives shared by several older participants, many of whom had never perceived racism during their health care visits but who had perceived its blatant expression in their everyday lives during their younger years:

*“I can remember that when I was a young girl… Going to school*,* you know*,* elementary school*,* I was called a name and I didn’t appreciate it. But I mean*,* at that time*,* a lot of the Black children were called… negress and nigger Black and things like that*,* you know? But as I grew older*,* you know*,* I didn’t have those experiences.”* – Allison, Woman, 79, Canadian-born.

Perceptions of racism in health care were also influenced by participants’ exposure to unique racial discourses arising from their personal immigration history. Participants who had previously lived in countries where overt racism is commonly perceived and named, as well as countries where race is not considered as an important social classifier, described difficulties in identifying manifestations of racism within their health care encounters in the Canadian context:

*“Coming from the UK*,* where systemic racism is so so apparent*,* evident*,* I refuse to see or believe that there is anything like that in Canada. So*,* maybe there is but*,* you know*,* when you’ve been culturally abused so much*,* any other racial abuse which is not as much as you’ve experienced in the past*,* to you it’s not racial abuse”* – Antoinette, Woman, 39, Immigrant 10 years or less.

*“You know*,* like*,* I came to Canada as an African… I didn’t know racism could exist. (…) You don’t pay attention to these things*,* like*,* you think they are not real. (…) Blackness is not a thing in Nigeria. Most people will just first of all see themselves as human beings and see themselves as Nigerians or wherever*,* whichever ethnic group they come from.”* – Robert, Man, 28, Immigrant 10 years or less.

Some participants shared that they might not have perceived racism during their health care encounters due to luck or because they do not seek care frequently and may therefore have had limited exposure. Meanwhile, others stated that they simply never considered it and therefore might not have noticed its expression.

### Theme 2. Ambiguous perceptions of racism in health care

After reflecting on the influence of racism in their everyday lives, some participants expressed uncertainty about their exposures to racism in health care, per se, due to their inability to compare their experiences to those of people from other racial groups or due to their discomfort in generalizing their personal experiences in this manner. For example, after having his concerns surrounding chest pain dismissed at an emergency room, one participant shared his disquiet in attributing his experiences to racism:

*“I can’t just point [to] her and say*,* ‘You’re a racist*,* you’re a racist! That’s why you’re impatient.’ I mean*,* you can’t just blame people like that; you don’t know their life. So*,* it’s hard to… Will she listen more if I was Caucasian? I don’t know.*” – Earl, Man, 35, Immigrant more than 10 years.

Many participants who expressed uncertainty about their experiences of racism in health care indicated an awareness of reports and media describing anti-Black racism in health care or of anti-Black stereotypes that hinder the ways in which Black patients are treated. Other participants shared anecdotal stories of their Black family members and friends who faced racism in health care. Such knowledge led some to recognize that racism may exist in health care and to question the quality of care they received. The potential influence of other social locations, like age and gender identity, further complicated perceptions of the causes of poor health care experiences, leading to uncertainty about the level to which racism impacted experiences:

*“Well*,* I’ve already asked myself like what his problem was*,* but I’ve never fixated on one particular point. You know*,* later*,* I thought that it was because I’m young. I wondered*,* maybe it’s because I’m a woman and maybe it’s because I’m Black. (…) In fact*,* for me*,* it’s as if he saw a young Black woman and then he just didn’t take me seriously or he thought I didn’t deserve to*,* you know*,* have a family doctor.” –* Anne-Marie, Woman, 25, Canadian-born (Original in French).

Other participants considered whether their poor experiences were caused by structural limitations like staff shortages resulting in providers having limited attention or genuine care, or other forms of discrimination, especially that which pertains to language vis a vis anglophones.

### Theme 3. Perceptions of overt interpersonal racism in health care

A minority of participants described experiences of blatant interpersonal racism, characterized by health care providers’ exhibiting racial prejudice or differential treatment practices, which led to reduced quality of care. One participant, for example, shared that a physician refused to touch her newborn daughter during her regular consultations, while another participant described being refused medication for a dermatological concern:

*“I’ve had acne as a teenager*,* and so I went to the dermatologist with my mother and*,* you know*,* I have friends who also had acne and had received medication*,* like topical anti-acne medication. (…) The acne was causing hyperpigmentation*,* so I asked [my dermatologist] if anything could be done about the dark spots*,* and he said*,* ‘Well you have to live with it*,* you’re Black.’”* – Sherry, Woman, 40, Canadian-born.

In another instance, stereotypes relating to the oversexualization of Black women (or the *Jezebel stereotype* which represents Black women as sexually deviant seductresses, and which reduces them to their bodies) impacted one participant’s perceived quality of care when seeking an abortion:

*“Once*,* when I had an abortion*,* and it was my first one and I was like 20-something (…) they treated me like it was my 16th and I was 14 [years old] or something. (…) I talked to a White friend that went and it was her third one and she was younger*,* and she never had anything like that*,* you know? [As a] Black woman*,* they were like*,* ‘you know*,* we’re not your contraceptives here blah blah blah blah.’* – Sarah, Woman, 43, Canadian-born.

### Theme 4. Perceptions of covert interpersonal racism in health care

Covert and subtle interpersonal manifestations of racism in health care were among those most frequently perceived. Most accounts dealt with physicians and nurses, but participants often emphasized that other health care workers, such as secretaries and technicians, are also implicated. Common covert manifestations are described below, along with their link to inequities in the provision of care:

### Downplaying of health concerns

The downplaying of health concerns and health care needs were commonly perceived as manifestations of racial discrimination. Several participants described feeling overlooked during their health care encounters – that their health concerns were being dismissed, and not receiving adequate attention, direction, or care. Such experiences were perceived to be less prevalent among providers with greater experience working with multicultural patient populations, or those who openly aim for “holistic” or “POC (person of colour)- friendly” approaches. Limited seriousness assigned to participants’ pain levels, or the failure to inquire about participants’ pain levels at all, resulted in longer wait times than some participants deemed normal or acceptable, or in the refusal of providers to perform clinical tests that participants believed would be helpful:

*“They didn’t really like take what I was saying seriously*,* in terms of how I was describing my symptoms to them. They were more like*,* you know*,* like sit down*,* we’ll get to you when we get to you. (…) They didn’t really want to run any more additional tests to see what was wrong with me. They kind of just were trying to send me home.”* – Ava, Woman, 22, Canadian-born.

For certain participants, varying social locations, such as age, immigration history, and gender identity, intersected with race to exacerbate their feelings of being dismissed during their health care encounters. For example, one younger participant was persistently denied tests under the presumption that she was too young to have an illness that she believed she had, resulting in her being initially misdiagnosed:

*“I noticed that I really had to be at death’s door for anyone to take me seriously. But if prevention had been in place*,* maybe it wouldn’t have gone so far. Also*,* from the very first time*,* [if] I was given the services I should have received*,* things wouldn’t get worse. That’s it. That’s it. It’s been like this for a long time. I was born here. It’s always a problem to be taken seriously (…).”* – Jada, Woman, 40, Canadian-born (Original in French).

Another participant was disregarded after being assumed to have immigrated to Canada illegally and of not having her required immigration documents:

*“For example*,* I was pregnant with my daughter. It was at the hospital that… because in fact it was a high-risk pregnancy*,* so I had to*,* for example*,* at least maybe once a week [go] to have blood tests (…). And well*,* the staff*,* she told me… Since I’m an immigrant*,* she said ‘Oh dear*,* she’s still coming for papers. (…)*’ – Tina, Woman, 37, Immigrant 10 years or less (Original in French).

For another participant, “performing illness” became a common mechanism aimed at reducing instances of dismissal:

*“I just feel like almost like I have to sort of perform illness in a way because as soon as they see me*,* they just see me as this Black dude and like the only Black guys that they… It seems to me that even in a place like Montreal*,* the perception is just young*,* Black men just either as athletes or delinquents or some weird combination of both and like*,* you know*,* I realized that when I come in and they just kind of give me that certain attitude*,* like I really just feel like*,* okay*,* they need to understand like*,* yeah*,* I really have this condition. So*,* I really try to be as specific [as possible] and I try not to sort of present like somebody who is*,* I don’t know*,* somebody who is in like perfect health or whatever. But it’s very hard.”* – Elijah, Man, 36, Canadian-born.

Despite such experiences, a minority of participants shared a sense of appreciation for Canadian health care when compared to experiences with foreign health care systems, implying that racism may be less of an issue in the Canadian context:

*“My husband is American and*,* when I was pregnant*,* I was debating if I was going to give birth in the States or in Canada. And because of the crazy mortality rates for Black women in the States*,* I was like there’s no way I’m giving birth there ‘cause at least I know that …I feel that it’s gonna be somewhat better here*,* you know? (…) Even if I don’t feel as considered and I also often feel dismissed by health care professionals here*,* actually like it’s even worse over there.”* – Sarah, Woman, 43, Canadian-born.

### Stereotyping in health care

Many participants discussed anti-Black stereotypes that impeded the quality of health care provided to them. These stereotypes were often presented in tandem with negative ideas that targeted other social locations like immigration history, gender identity, and age. For example, stereotypes related to inappropriate drug use were raised by some of the young men in the study, several of whom perceived that such ideas led health care providers to not believe their health concerns and, in some cases, to withhold prescribing certain medications:

*“I remember one time*,* I was pretty sick*,* and I went to the ER and I was triaged by a nurse. And I wasn’t feeling good in between*,* and she was like*,* ‘I’m not gonna give you pills. I’m not gonna prescribe you any pills.’ Like I never asked for pills one time*,* and she was pretty mean and aggressive. I think that was my first experience where I felt racialized in the [health care] environment.*” – Andrew, Man, 26, Canadian-born.

On the other hand, a few participants, particularly those of Caribbean heritage, described having faced preconceptions that Black patients are dependent on medications. They recounted experiences during which health care providers doubted their accounts that they were not taking any prescribed medications due to assumptions that they must have a chronic illnesses like diabetes or hypertension.

Racial and gendered stereotypes also intersected, impacting the care that Black women reported. Several Black women shared that beliefs that Black women are strong and have higher pain tolerance reduced the quality of care that they received as they are often treated as though they are exaggerating their symptoms:

*“We believe that*,* well*,* to a large extent*,* that the Black woman has no pain. She is a superhero. She doesn’t suffer. Or*,* we say that she exaggerates because*,* in fact*,* we’re supposed to handle pain easily. But pain isn’t a question of [skin] color.”* – Tina, Woman, 37, Immigrant 10 years or less (Original in French).

*“I was praised*,* you know*,* for having the surgery without anesthesia and ‘oh you took it so well’*,* ‘why do you think you took it so well; do you think it’s hereditary?’ You know*,* Black women are assumed to be strong. You know*,* the ideology of the Black strong woman still resonates with a lot of people; that we’re tough*,* that we’re*,* you know…”* – Sherry, Woman, 40, Canadian-born.

Some participants recounted facing infantilization, dismissal, or underservice as a consequence of their providers assuming that Black patients lack the literacy or intelligence to understand and adequately describe their health needs. Such preconceptions were often manifested at the intersections of race, immigration history and age. For example, having a foreign accent or struggling with French (the official language of the province of Quebec) sometimes resulted in immigrants being treated as “undesirable clientele” who do not have the capacity to appropriately describe their needs. Ageism was also perceived by several older participants who recounted feeling as if they were not being listened to:

*“[My father] was in a lot of pain and he was telling [the nurses] that he had been feeling for a few days that his stomach has been distended. (…) He’s describing*,* you know*,* really very technical terms what he’s been feeling and his symptoms. And the nurse keeps ignoring him and saying ‘Monsieur*,* monsieur… your tummy*,* your tummy hurts? Your tummy is here.’”* – Sherry, Woman, 40, Canadian-born.

One participant added to this idea by describing how ableist ideas may also exacerbate negative preconceptions relating to race and age:

*“My mother was also a very sick person*,* but she was paralyzed. But as I was the person who had to speak for her*,* I had to be as professional as possible because*,* otherwise*,* not only did they not listen to her*,* but they didn’t do what she needed. They spoke to her really badly. And that’s another problem*,* people who are physically disabled*,* we often think they’re mentally disabled*,* whereas that’s completely false.” –* Jada, Woman, 40, Canadian-born (Original in French).

### Racial microaggressions

A racial microaggression is defined as “the everyday slights, insults, putdowns, invalidations, and offensive behaviors that people of color experience in daily interactions” by persons who may be aware or unaware of the offensive treatments towards the target groups [77]. Many participants described experiencing racial microaggressions in health care, making their inequitable treatment more difficult to identify or confront. The subtlety of these expressions in the Canadian context was sometimes presented in contrast to the United States, where racial injustices are often shared in popular media outlets and racial discourse is more commonplace. Several participants explained that the subtle nature of such forms of discrimination may render it difficult to reveal, and may result in its acceptance or normalization, as well as a disempowerment for them to attempt to “do something about it”:

*“It’s very difficult to like describe these things also because they’re so like intangible. It’s just like a feeling that you get but also because it’s happened so often that like I really just kind of don’t think about it. Like you kind of just put it away in your mind and you basically like… I would say I’ve been sort of conditioned to really just focus on the things that I can control. And people’s attitudes and stuff… I mean there’s very little that I can do about that so…”* – Elijah, Man, 36, Canadian-born.

The subtle nature of racism in the Canadian health care context led to some participants being hypervigilant and to question different ways in which their race works alongside other factors to result in their disparate treatment:

*“It’s hard to be really specific because*,* remember*,* you’re on your guard all the time. So*,* it’s hard to be specific to say*,* ‘Hey*,* this happened. Did it happen because of my race or did it happen because the person just had a bad morning?’ You know*,* it’s really hard to… Unless it’s directly in your face*,* it’s hard to tell whether it happened because [of] your race*,* but I can say that most of the time things happen to you*,* to me at least*,* that I don’t like*,* it’s because of my race or the French. Because I know I don’t do anything to aggravate them*,* I know that…”* – Jackson, Man, 70, Immigrant more than 10 years.

Comparing the Canadian context to that of the USA, some participants expressed frustration with the lack of acknowledgement made to racism in Canada:

*“Today*,* it’s hard to say: ‘Yes*,* it’s because we’re Black’*,* you know? But*,* especially in Quebec*,* there are laws*,* so people do it subtly. They’re not going to do it openly because of course there are laws. So*,* they’re going to do it subtly and stuff*,* we could say it’s more like microaggressions.”* – Tina, Woman, 37, Immigrant 10 years or less (Original in French).

*“We talk a lot about the United States*,* but there’s a lot of things going on here too. Except that*,* here*,* things are more hidden*,* coated*,* people are less direct. People are less direct*,* but there’s still a problem.”* – Jada, Woman, 40, Canadian-born (Original in French).

One participant described subtle racism as being potentially more harmful than overt racism as its insidious nature makes it more difficult to confront:

*“When it’s subtle racism*,* you have to be careful… (…) I would more trust the racism that’s in your face*,* so you know where that person is coming from*,* than to have the same situation but it’s subtle. You know? So… These little subtle things*,* sometimes*,* they can hurt you worst than out-there racism*,* if you know what I mean… you know…?”* – Shanice, Woman, 68, Immigrant more than 10 years.

### Theme 5. Perceptions of systemic racism in health care

Many participants described the inability to disconnect or to distinguish between racism that is manifested within Quebec more generally, and racism that exists within health care in Montreal, more specifically, explaining that health care workers bring biases rooted in prior norms, images, and experiences:

*“Prejudices comes at a very young age in relation to parents and the educational makeup*,* in fact. So*,* for me*,* it’s like I say*,* whether it’s a doctor or someone*,* anyone*,* even outside the medical system*,* their prejudices are there.” –* Tina, Woman, 37, Immigrant 10 years or less (Original in French).

*“People say they’re neutral*,* but they’re not in reality. Just because you’re a doctor doesn’t mean you’re neutral. It’s not true. You’re a human being first.*” – Jada, Woman, 40, Canadian-born (Original in French).

Some participants expanded on this idea by explaining that social hierarchies and accompanying systems of power and privilege may expose Black people to greater opportunities for racial discrimination in health care. Such participants recounted that class, for example, may intersect with race to influence Black patients’ perceived value in the health care environment:

*“Sometimes*,* it’s not easy to pinpoint*,* but you definitely know [that] if you were somebody else with more power and more [resources] (…)*,* I don’t think this would happen [to you] (…). Power is that… yes*,* you’re following a group of people that no one would…like people would be really cautious to treat you differently because they know [that] you could take it serious and they could lose their… like you could file a complaint against them. Like*,* they wouldn’t even dare to treat you that way because they wouldn’t be able to recover from the damages that would result.”* – Robert, Man, 28, Immigrant 10 years or less.

Such differences in class, power and privilege may also impact interpersonal exchanges between persons assigned to the same racial group, further complicating racial dynamics in health care. For example, one participant describes having received poor health care from a racially concordant physician due to what she perceived as their internalization of anti-Black racist ideas, leading them to give greater importance and positive stereotypes regarding power and resources to White persons:

*“I think systemic racism*,* you know*,* it affects Black communities as well. It affects our interactions amongst each other. And I think that*,* had I been White*,* [my Black family physician] would have been afraid of mistreating me or not listening to me because he might have felt like White people might have access to resources that Black people don’t have access to*,* or they might have a bigger voice than Black people have. He might have thought that I probably wouldn’t have said anything*,* you know… and I didn’t.”* – Megan, Woman, 37, Canadian-born.

Other participants referred to Quebec’s culture around the French language when describing ways in which prejudice against the English language may intersect with racial prejudice to exacerbate experiences of racial discrimination in health care:

*“Especially as a POC who’s English*,* like anglophone… I mean*,* I speak French but*,* at the end of the day*,* like I prefer to speak English*,* and I find that going to hospitals in Quebec… I mean*,* it’s not every nurse but a lot of them*,* as soon as you kind of start speaking to them in English*,* it’s like a problem. It’s like they have this idea*,* of ‘Oh*,* it’s Quebec*,* you have to speak French.’ So I don’t know if maybe that plays into it too. It’s like ‘Oh*,* you’re Black and you don’t speak French.’”* – Ava, Woman, 22, Canada-born.

*“And then there’s the language barrier. Me*,* I don’t have problems there*,* but I know that those who have problems speaking either French or English… It all depends on which hospital they go to*,* because it’s clear that if they go to a francophone hospital and they only speak English*,* they’ll be discriminated against*,* and if they go to an anglophone hospital where people don’t speak French*,* the same thing happens. Even personally*,* I was more welcome in an anglophone hospital than a francophone one. (…) I don’t know if it’s open-mindedness or not. I don’t know at all*,* but I’ve changed hospitals (…) and since I’ve gone to an English-speaking hospital*,* I haven’t had any problems. (…) I felt more welcome. More welcome with less prejudice [than] the francophone hospital.* – Tina, Woman, 37, Immigrant less than 10 years (Original in French).

## Discussion

This study contributes novel empirical insights into how Black patients do, or do not, perceive racial discrimination in health care, and demonstrates how anti-Black racism and other systems of power might combine to create unique health care experiences among those who do perceive racism during their health care encounters. We identified five major themes that reflect degrees of perceived racism in health care among Black adults: (1) no perceptions of racism in health care, (2) ambiguous perceptions of racism in health care, (3) perceptions of overt interpersonal racism in health care, (4) perceptions of covert interpersonal racism in health care (including the downplaying of health concerns, stereotyping, and racial microaggressions), and (5) perceptions of systemic racism in health care.

Our findings point to important variations in perceived racism and racial discrimination among Black persons in health care. Participants’ perceptions of racism and racial discrimination were often shared in reference to past experiences of racism in health care, in other institutions, or within their everyday lives, or to particular racial discourses in Canada and other geographical regions. For example, the absence of any perceptions of racism in health care in Montreal was commonly described by participants with limited exposure to Canadian racial discourses and among participants who reported experiences of overt forms of racism in other contexts. Participants also often interpreted racism in their health care experiences based on their knowledge of systemic forms of racism in Canada and other countries, including the long history of racial health care disparities in the USA. These findings resonate with Essed’s description of strategies used by individuals to interpret and evaluate racial discrimination, which includes inference from beliefs, expectations and knowledge about racism, and comparisons to one’s own experiences or the experiences of others for (in)consistency and consensus [78]. Essed posits that those who perceive experiences of racism deploy rational evaluations and argumentations, and that their interpretations are not ad hoc. As such, she maintains that the perspectives of those who face greater experiences of racism may thus be more relevant in identifying contemporary manifestations as their accumulated experiences and understandings render them more expert [79].

In a similar manner, variations in attributions to anti-Black racism in Canadian health care generally, and health care in Quebec more specifically, may relate to differences in knowledge of historical representations of racism in the country and province, to differences in understandings of how racism may manifest beyond blatant interpersonal expressions, or to different meanings ascribed to attitudes and behaviours perceived as racist [80, 81]. Several quantitative studies have also signaled variations in perceptions of racism among Black groups as it relates to factors such as neighbourhood- and individual-level socioeconomic position, gender identity, education, income, immigration history and type of discriminatory behaviour [82–86]. For example, Husbands and colleagues found that Black people living in Canada were more likely to report experiences of racism if they were Canadian-born, employed, or had higher levels of education [86]. These complexities point to the need to consider both blatant and subtle forms of racism in efforts to address health care inequities.

Of particular note are participants who perceived no racism in health care. While this may be true, questions of denial or the rationalization of prejudice through processes of internalized racism should also be considered [87, 88]. Also at play is the rhetoric of national exceptionalism which distinguishes between historical and contemporary racial issues in Canada and the USA, portraying Canada as a multicultural state, free of racism and intolerance [89–91]. As a result of this rhetoric, experiences of racism in Canada might be even more covert and insidious [92], and more difficult to identify and to confront. Some participants’ positive outlook on Canadian health care services when compared to the USA also suggests that Canada’s dearth of literature related to inequities in health and health care might be treated as evidence that inequities do not exist. This perception highlights the need to build a larger evidence base on race and health in the Canadian context, which would begin with the collection of national quality health data that is disaggregated by race.

Our study findings further indicated variations in specific attitudes and behaviours that are perceived as racist or racially discriminatory. Supporting the existing literature [36, 93, 94], such expressions included both covert and overt forms of interpersonal racism, as well as perceptions of systemic expressions. Interpersonal manifestations described by our participants mostly included covert expressions such as the dismissal of their healthcare needs, an indifference to the seriousness of their pain levels, prejudice based on negative Black stereotypes, and racial microaggressions. A minority of participants described overt expressions of racism such as differential health care treatment based on race and avoidance of provider-patient physical contact. Others expressed perceptions of systemic racism as it relates to the privileged status of the French language in Quebec, and to the normalization of racial prejudice and discriminatory behaviours across various institutions and contexts within society at large, including in health care. These findings emphasize the importance of considering various mechanisms through which anti-Black racism and discrimination may be perpetuated in health care, particularly within the context of Quebec where the presence of systemic racism has been openly denied by leading government officials [95, 96]. As perceptions of racism in health care have been associated with delays in seeking care and unmet health care needs [97, 98], strategies that counter anti-Black racism and discrimination might directly improve care experiences and help to redress racial health inequities faced by Black communities.

Participants’ narratives also revealed intersectional complexities in their experiences of racism and racial discrimination in health care. In keeping with intersectionality’s contention that social identities and statuses are not additive but mutually constitutive [99], participants’ narratives often illustrated the importance of recognizing the multiplicity of their social locations in shaping experiences of anti-Black racism. For example, when sharing feelings of being dismissed or being subject to racial stereotypes in the health care setting, several participants referred to more than one identity, such as “Black woman”, “Black man”, or “young Black man”- Black women referring to racial stereotypes regarding higher pain tolerances, and Black men to assumptions that they are athletic and in good overall health. These stereotypes resulted in both groups feeling overlooked in health care. The influence of particular social locations, and their related systems of power, on health care experiences are not limited to those discussed in this study and may also include, for example, impacts of socioeconomic status and ethnicity. As argued by Bowleg [100], such narratives highlight intersectionality’s assertion that an individual social category (such as race) should not be the sole focus in investigations of social differences. As evident in our study, a singular focus risks obscuring richer understandings made evident through considerations of how race intersects with other social locations such as gender identity, age, and language.

Intersectionality’s central principles also assert that individual experiences related to particular social locations reflect macro-level systems of power and social inequalities [71, 100, 101]. Consistent with this tenet, participants’ narratives indicate how individual social locations, such as race, gender identity, age, and immigration history, intersect in ways that reflect micro- and macro-level systems of dominance, such as racism, sexism, ageism, and xenophobia. Indeed, we noted instances where perceived racism intersected with other forms of perceived discrimination to shape participants’ experiences with health care. For example, some of our older participants shared experiences of both racism and ageism (as it relates to their racialization as Black and older age) when seeking health care, signaled through their treatment as incapable of adequately understanding and describing their health care needs and, consequently, being infantilized or dismissed. Many women in our study reported facing gendered racism (that is, interconnected experiences of racism and racist constructions of gender expectations) [102], describing how negative stereotypes of the “strong Black women” or the “Jezebel” hindered the quality of care that they received. Several men in our study also reported facing gendered racism though descriptions of how negative stereotypes regarding delinquency and drug use affected their health care encounters. Finally, one participant discussed ways in which racism, ageism, and ableism interlocked to impede the quality of care received by her mother due to her racialization as Black, older age, and physical disability. These findings contribute to an emerging literature describing intersectional experiences of health care, and demonstrate the unique barriers faced by patients that are marginalized at multiple levels related to race and other disadvantaged social locations [103–106].

## Implications

Although interest in, and engagement with, intersectionality approaches in public health has grown in recent years, studies that explore the influence of multiple, interconnected systems of privilege and disadvantage on health disparities remain relatively scarce [73]. Our research findings suggest various ways in which perceived racism, and other systemic and intersectional factors, shape the health care experiences of Black patients. Since access to quality health care is crucial in achieving equitable health outcomes, our findings have important implications for public health and health policy. First, our findings emphasize the need to incorporate more complex understandings of racism and racial discrimination in health care policy, including those related to training and service delivery. Such considerations would highlight the need for multiple, tailored approaches that address anti-Black racism through interventions targeting specific attitudes, behaviours, and decisions at different levels of healthcare systems. Equitable access to health care, and associated improvements in racial health disparities, require inclusive policies that recognize and understand the complexity of racial issues, and which are not dependent on ad hoc measures or individuals working alone [107]. Our study also highlights the potential for intersectional programming that recognizes the multiplicity of social locations and related systems of power that impact the health care experiences of Black patients. These programs might incorporate systemic and institutional analyses of racism and facilitate a movement away from cultural competency in health care education and practice, to anti-racist education and practice in health care, to address the root causes of racial health disparities [25, 108]. One example is the development of medical school curricula that makes explicit how various forms of racism and interlocking systems of disadvantage shape health care access and experiences to ultimately produce health inequities [109]. Notably, relevant initiatives are already underway, such as the Black Health Education Collaborative’s *Black Health Primer*, a program designed to fill knowledge gaps in training and education on Black health and anti-Black racism in Canadian medicine and public health [110]. Health care associations in Canada, such as the Canadian Nurses Association and the Collège des médecins du Québec, have also demonstrated commitments to acting against racism in medical care [111, 112]. Explicitly naming anti-Black racism and describing its effects on health through an intersectionality lens is an important step towards building and maintaining equitable health care systems. Such an approach may also facilitate health care providers’ considerations of broader social and structural factors that influence health beyond the level of the individual, allowing for more holistic assessments of patient needs.

Beyond health care, our study also reinforces the importance for public health researchers, practitioners, and decision-makers to directly recognize and confront racism as a public health concern [113]. Racism is a fundamental cause of health and illness, driving racialized trends in morbidity and mortality. As the central role of public health practitioners includes the promotion of health and the prevention of illness and premature death for all persons, it is imperative that public health actively incorporates anti-racist work [114]. To achieve optimal health for all, public health practitioners must recognize societal factors driving racialized health disparities (including racism and racial discrimination) and take action to avoid their perpetuation and exacerbation. Finally, our study also suggests the importance of considering racism as a structural determinant of health in the development of broader public health policies and in health education. It highlights the importance of confronting the socio-political context of population health and emphasizes that policies aimed at achieving health equity cannot assume a one-size-fits-all approach. Diverse contextual factors at both micro and macro levels of society must be considered in interventions aimed at addressing racial health inequities at the population level. Further, public health actions aimed at addressing anti-Black racism should implement cross-sectoral strategies that account for intersectionality, and that target policies and practices spanning beyond the health sector that have been shown to impact the determinants of health, such as those relating to education, welfare and social protection, and culture and media [115]. Such intersectoral actions may facilitate the development of more collaborative and responsive health interventions that redress racial health inequities through tailored strategies.

## Limitations

Due to its qualitative design and intention, findings from this study should not be generalized to other settings. It is also worth noting that the ethno-racial diversity of Montreal is not necessarily mirrored elsewhere in the province of Quebec and across other regions in Canada [116, 117]. Perceived experiences of racism and racial discrimination in health care may differ in regions with different demographic compositions, particularly as it relates to ethno-racial diversity. Also, while data analysis was undertaken by a single researcher, several strategies were employed to mitigate bias, such as regular debriefing meetings between members of the research team and the use of a data matrix which facilitated the identification of themes across participants. Finally, our sampling strategy relied on two main avenues which may have narrowed the diversity of participants: social media or affiliation with a community organization or academic association serving people who identify as Black, Caribbean and/or African. We mitigated this limitation by implementing a maximal variation sampling approach which intentionally set about recruiting diverse participants.

## Conclusion

Perceptions of anti-Black racism and racial discrimination in Canadian health care are complex, reflecting the heterogeneity of Black populations and the intersectional nature of such experiences with respect to social locations and micro and macro level systems of power. Participants’ unique narratives demonstrate that perceptions of anti-Black racist attitudes and discriminatory behaviours in health care should not be summarily generalized to Black populations without consideration given to potential intra-racial group differences. While some participants reported absent or ambiguous perceptions of racism in their health care experiences, others shared narratives detailing how their particular social locations cut across various axes of marginalization to create unique encounters that they perceived as racist. It is therefore crucial to consider patients’ experiences in investigations of health care quality and accessibility, as the outcomes of their health care encounters may be very different despite commonalities in the system or facility where care is sought. Advancing racial health equity requires greater sensitivity from providers and decision makers to variations in Black patients’ experiences, and corresponding measures to ensure access to responsive, high quality health care services.

## Electronic supplementary material

Below is the link to the electronic supplementary material.

Supplementary Material 1

Supplementary Material 2

## Data Availability

No datasets were generated or analysed during the current study.

## Abbreviations

POC: Person of colour
