# Supplementary material for: Anti-Black racism in Canadian health care: a qualitative study of diverse perceptions of racism and racial discrimination among Black adults in Montreal, Quebec
Source: BMC Public Health. 2024 Nov 13;24:3152. doi: 10.1186/s12889-024-20636-0 (PMC11562605; doi:10.1186/s12889-024-20636-0)
Supplement: Supplementary file 1 — Supplementary Material 1 [file 12889_2024_20636_MOESM1_ESM.docx]

Additional File 1. Interview Guide (English)

| **Main Questions** | **Probes and follow up questions** |
| --- | --- |
| How do you feel you’ve been treated as a patient in the times that you’ve sought healthcare? | - Please describe an experience.   - Where did this take place?   - Who was involved? - Were you satisfied?   - Please describe an example. - Have you ever had the feeling that you should have been treated better?   - Please describe an example. - [Probe for impact of intersectionality]:   - Education? Immigration? Age? Culture? Income? Occupation? Living situation? Community? Religion? |
| Have you ever felt that you are treated differently by members of healthcare systems (i.e., hospitals or clinics that you seek consultations from) because you are Black? | - If yes:   - Tell me about these experiences.   - Good experience?   - Difficult experience?   - Who was involved in this experience?   - Where did this take place (e.g., family medicine clinic, ER)?   - Feelings?   - Response? - Have you felt the need to modify how you present yourself while seeking healthcare (e.g., your appearance; the way you speak)?   - Why or why not?   - If yes: Please describe an experience.   - Feelings? - How often do you interact with healthcare staff that are racialized?   - Are they Black or a different race?   - How does this impact your experience with seeking healthcare services? |
| Tell me about an experience that made you aware of your race? | - Describe a time when you felt people treated you differently because of your skin color:   - Treated better than others?   - Treated worse than others?   - Where (e.g. workplace, various services, personal life, healthcare)?   - Feelings?   - Response? |
| To understand your relationship with the healthcare system and your ability to access healthcare services, I would like to ask you a few questions concerning your healthcare needs. Firstly, how was your health before the pandemic? | - How often did you see a doctor?   - Type of doctor?   - Multiple providers? - Do you have a family physician? If yes:   - How many times did you see a family physician?   - Please describe your experiences.   - Satisfied with the services you get? Why or why not? - Did you ever go to the emergency room?   - If yes:   - What made you decide to seek a consultation?   - Did you attempt to consult with a family physician first?   - Good experience? Bad experience?   - If no:   - Did you consider going to the ER?   - Why didn’t you go? |
| How has your health been during the pandemic? | - Did you test positive for COVID-19? - Did you have any healthcare needs unrelated to COVID-19? If yes: Did you consider scheduling a consultation?   - If yes: Did you manage to see a doctor? Please describe your experience. Any difficulties? Any facilitators?   - If no: Why not? |
| What could a safe and accessible healthcare service look like for the Black community? | - What do you value in terms of the healthcare services that you have received? - Do you believe primary healthcare should be improved for the Black community?   - If yes: How (e.g. rules, policies, accommodation)?   - If no: Why not? What attributes are important to you? - How would you describe a perfect healthcare consultation?   - What can physicians do?   - What can nurses do?   - What can other employees (such as secretaries and security guards) do? |
| Any other thoughts or concerns that you would like to share? | N/A |
